# Increase in anthelmintic resistance in sheep flocks in Ireland

**DOI:** 10.1186/s13620-025-00317-z

**Published:** 2026-01-22

**Authors:** Kyra M. Hamilton, Padraig O’Boyle, Amanda McEvoy, Dave M. Leathwick, Theo de Waal, Francis Campion, Orla M. Keane

**Affiliations:** 1 Animal and Bioscience Department, Teagasc Mellows Campus, Athenry, Co Galway Ireland; 2 Grasslands Research Centre, AgResearch, Palmerston North, 4442 New Zealand; 3 School of Veterinary Medicine, University College Dublin, Dublin 4, Ireland; 4 Inparacon Ltd, Leeston, New Zealand; 5 Animal and Bioscience Department, Teagasc, Grange, Dunsany, Co. Meath Ireland

**Keywords:** Anthelmintic resistance, Sheep, Ivermectin, Benzimidazole, Levamisole

## Abstract

Gastrointestinal nematode (GIN) infection is widespread in grazing lambs in Ireland, with heavy infections resulting in ill-thrift and ill-health. Control is currently dependent on the use of broad-spectrum anthelmintics. However, both anthelmintic resistance (AR) and treatment failure have been reported in Irish flocks. Most recent reports have determined the rate of treatment failure. Therefore, to ascertain the level of AR on sheep farms in Ireland 77 faecal egg count reduction tests (FECRT) were carried out on 24 farms between 2018 and 2023 with the test repeated on 3 farms in another year. Resistance was widespread, with all farms showing evidence of resistance to benzimidazoles. For levamisole, 50% of farms evidenced susceptibility and 42% resistance; the remaining 2 farms had conflicting results in different years. For the macrocyclic lactone, ivermectin, susceptibility was found on 25% of farms and resistance on 71%. The remaining farm had conflicting results in different years. Moxidectin was tested on 3 farms and all 3 showed evidence of resistance. For a small number of tests larvae were identified to genus level pre- and post-treatment. The predominant genus post-treatment was *Teladorsagia circumcincta* although *Trichostrongylus* spp. and *Cooperia* spp. were also identified. Overall, AR in GIN was widespread on sheep farms in Ireland and the adoption of sustainable parasite control practices that reduces reliance on anthelmintics is imperative. The finding of conflicting results in different years highlights the importance of regular monitoring for AR and the need to incorporate GIN species identification into the FECRT to enable species-specific efficacy calculation.

## Introduction

In recent years there has been a number of reports of anthelmintic resistance (AR) or anthelmintic treatment failure against gastrointestinal nematodes (GIN) of sheep in Ireland. The first report of resistance dates to the early 1990 s and concerned resistance to the benzimidazole class of anthelmintics [14]. Since then, studies have quantified resistance [4] or treatment failure [7, 9]. Faecal egg count reduction tests (FECRTs) conducted between 2002 and 2010 on 18 Irish sheep farms evidenced resistance to benzimidazole and levamisole in 88% and 39% of flocks respectively [4]. Resistance to ivermectin was not tested but an in vitro micro-agar larval development test showed suspected resistance in 11% of flocks. The Sheep Technology Adoption Programme (2013–2015) tested treatment failure, based on the faecal egg count reduction (FECR) in pooled samples pre- and post-treatment. In 2013, failure of 70%, 48% and 24% of benzimidazole, levamisole and macrocyclic lactone treatments respectively was reported [7]. Over the full 3 year programme (2013–2015) failure of 69%, 48%, 37.5% and 16% of treatments with benzimidazole, levamisole, avermectins and moxidectin respectively were reported [9]. Even more worryingly, in 2015 two multi-drug resistant isolates of *Teladorsagia circumcincta,* resistant to benzimidazole, levamisole and ivermectin were described; the first report of multi-drug resistant GIN in Ireland [8]. These data suggest an increase in AR in sheep flocks in Ireland since the initial report of resistance; however recent data is based on pooled faecal samples rather than the gold-standard FECRT (Coles et al., 1992). Therefore, the extent of resistance was quantified using the FECRT on 24 sheep farms in Ireland between 2018 and 2023.

## Materials and methods

FECRTs were conducted on 24 farms between 2018 and 2023. The farms were located in Leinster (*n* = 9), Munster (*n* = 3), Connacht (*n* = 9) and Ulster (*n* = 3). On 3 of the farms FECRTs were conducted in 2 separate years. Between 2 and 4 anthelmintics were tested per farm, resulting in 77 FECRTs in total. Teagasc sheep specialist advisers identified clients appropriate for completing a FECRT. These farmers were contacted and invited to participate in the project and interested farmers self-selected. On all farms that completed the FECRT in 2018, lambs were treated with monepantel (Zolvix, Elanco, 2.5 mg/kg) at weaning to ensure any GIN that survived previous anthelmintic treatments were eliminated. However, this did not occur in subsequent years. Flock faecal egg count (FEC) was monitored every 2 weeks from weaning using the FECPAK^G1^ method with a multiplication factor of 30 (www.techion.co.nz). When flock FEC exceeded 400 eggs per gram (epg) the FECRT was conducted according to World Association for the Advancement of Veterinary Parasitology (WAAVP) guidelines [6]. On each farm up to 16 lambs per anthelmintic to be tested were randomly selected and weighed. Faecal samples were collected *per rectum* from each lamb. Lambs were then treated with the anthelmintic to be tested: fenbendazole (Zerofen, Chanelle 5 mg/kg), levamisole (Chanaverm, Chanelle, 7.5 mg/kg), ivermectin (Oramec, Merial Animal Health 0.2 mg/kg) or moxidectin (Cydectin, Zoetis, 0.2 mg/kg). All lambs were treated orally and to the weight of the heaviest lamb in the group being tested. The farm was re-visited 7–14 days post-treatment and faecal samples again collected *per rectum*. Faecal samples were stored at room temperature in plastic zip lock bags during transport to the laboratory. The required mass of faeces for FEC testing was removed and stored at 4 °C until analysis. The remainder of the faeces was put into larval culture as described previously [10]. The FEC of each faecal sample was determined using the mini-FLOTAC method (*n* = 59 tests) or the McMaster method with a multiplication factor of 25 (2 slides; *n* = 14 tests) or 50 (1 slide; *n* = 4 tests) as described previously [3, 12]. Only lambs with data both pre- and post-treatment were retained for analysis and any lamb with a FEC of 0 epg pre-treatment was removed from the results calculation. The aim was to have a minimum of 200 eggs counted pre-treatment [5]. Data were analysed using https://www.fecrt.com/ according to WAAVP guidelines [6] with an expected efficacy of 99% and a lower efficacy threshold of 90%. Results were classified as described below:

- Susceptible (S): the lower 90% CI ≥ 90% and upper 90% CI ≥ 99%
- Resistant (R): the upper 90% CI < 99% (includes Low Resistant with the additional criterion that the lower 90% CI ≥ 90%).
- Inconclusive (I): the criteria for neither R nor S are met.

For some tests, at least 50 larvae were identified pre- and post-treatment to genus or species level either morphologically according to the keys of [16] or by PCR. For PCR individual larvae were picked into the wells of a 96 well plate containing 10 µl of 0.5 X DirectPCR lysis reagent (Viagen) and 0.5 µl recombinant Proteinase K (Roche) and lysed at 55 °C for 16 h followed by 90 °C for 1 h. PCR was carried out in 1 X Platinum Green Hot Start PCR Mastermix containing 0.2 µM each primer and 1 µl of lysate (diluted 1 in 2). The reactions were activated at 95 °C for 3 min followed by 12 cycles of 94 °C for 10 s, 60 °C for 15 s (reducing by 0.5 °C per cycle) and 72 °C for 30 s then 25 cycles of 94 °C for 10 s, 54 °C for 15 s and 72 °C for 30 s followed bv 72 °C for 7 min. For each lysate 3 PCR reactions were performed with generic primers ITS2GF and IST2GR along with the species-specific primer for *Haemonchus contortus*, *Teladorsagia circumcincta* and *Trichostrongylus axei* (PCR 1); *Trichostrongylus colubriformis* and *Trichostrongylus vitrinus* (PCR 2) and *Chabertia ovina*, *Cooperia curticei* and *Oesophagostomum venulosum* (PCR 3) as per [2]. PCR products were visualised on a 2% agarose gel.

## Results

### FECRTs

The number of farms on which FECRTs were completed for each anthelmintic is shown in Table 1. For all tests the requisite minimum total number of eggs counted (i.e. the cumulative number of eggs counted before the application of the multiplication factor) recommended by WAAVP was reached [6].

**Table 1:** Number of farms on which a faecal egg count reduction test (FECRT) was conducted with each anthelmintic class

Anthelmintic active	Number of farms with completed FECRT	Number with tests repeated in another year
Fenbendazole	19	1
Levamisole	24	3
Ivermectin	24	3
Moxidectin	3	0

**Fig. 1:**
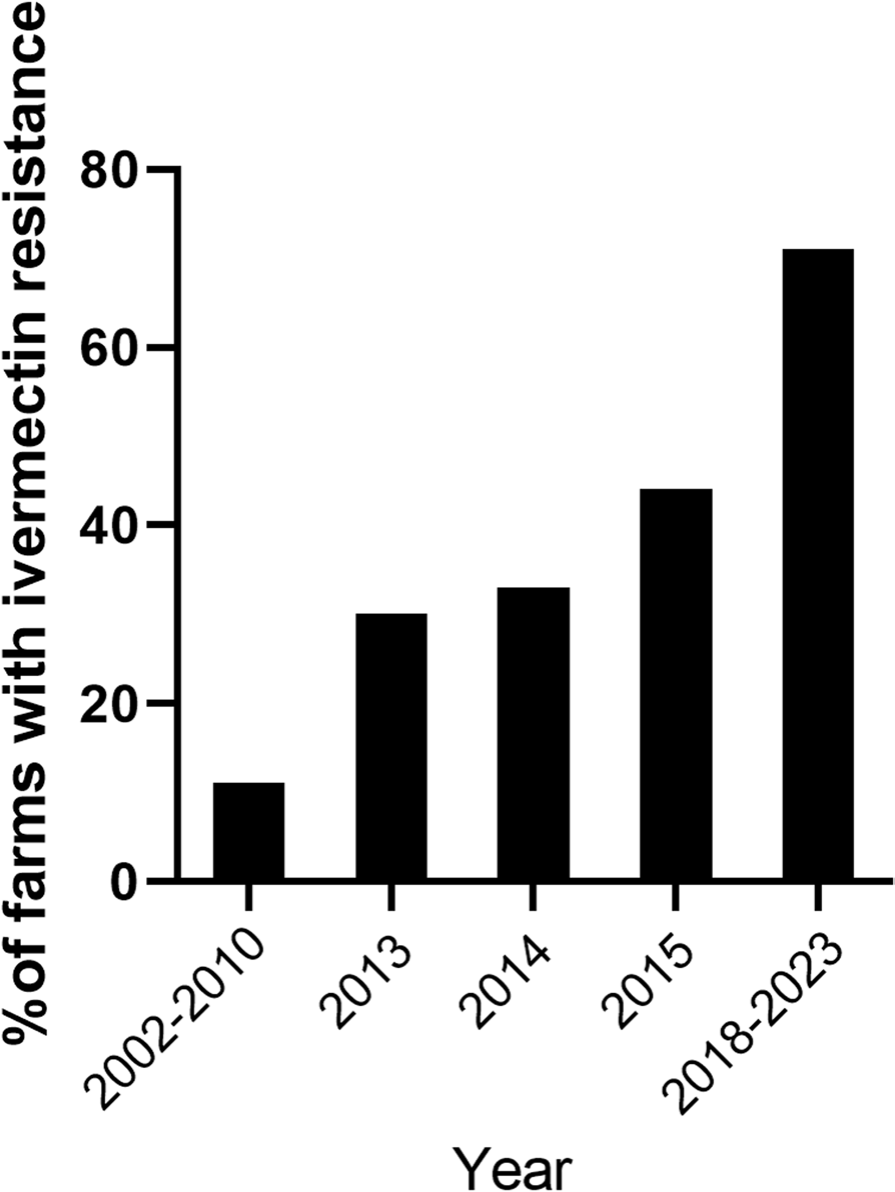
Putative ivermectin resistance based on in vitro larval development (2002–2010), testing of pooled faecal samples (2013–2015) or faecal egg count reduction tests (2018–2023) in sheep flocks in Ireland

The efficacy of benzimidazole was evaluated on 19 farms. On all farms benzimidazole resistance was evident with FECR ranging from 21.3% to 87.2%. The one farm on which the test was repeated after 3 years showed resistance in both tests. Levamisole efficacy was evaluated on 24 farms, with the test repeated on 3 farms in a later year. FEC reductions ranged from 61.4% to 100%. Susceptibility to levamisole was found on 12 farms, while resistance was found on 10 farms, of which 3 were classified as low resistant and one was classified as resistant in 2 tests. For the remaining 2 farms, on which the test was repeated, the results were conflicting in different years. Farm 7 was classified as low resistant in 2018 but susceptible in 2022 while farm 21 was classified as susceptible in 2018 and low resistant in 2022. The efficacy of ivermectin was also evaluated on 24 farms with the test repeated on 3 farms in a later year. FECR ranged from 49.5% to 99.8%. For 6 of the farms susceptibility was found with 17 farms having resistance, which included one farm that was classified as low resistant and 2 farms that were classified as resistant in 2 tests. The final farm (Farm 21) was classified as susceptible in the initial test in 2018 but low resistant in the subsequent test in 2022. Results of all FECRTs with benzimidazole, levamisole and ivermectin are shown in Table 2. Putative ivermectin resistance in Irish sheep flocks based on studies conducted in 2002–2010, 2013, 2014 and 2015 are compared to the results of the faecal egg count reduction tests in this report (Fig. 1).

**Table 2:** Results of the faecal egg count reduction tests carried out on 24 farms in Ireland between 2018 and 2023

		**Fenbendazole**				**Levamisole**				**Ivermectin**			
**Farm No**	**Year**	**FECR**	**90% U.I.**^**b**^	**Class**^**c**^	**Larval ID**^**d**^	**FECR**	**90% U.I**	**Class‡**	**Larval ID**	**FECR**	**90% U.I**	**Class‡**	**Larval ID**
1	2018	21.3	−16.3–52.5	R		99.0	98.4–99.5	S		96.4	90.5–99.6	S	
2	2018	69.6	45.6–87.3	R	Te, Tr^f^	95.8	92.4–98.4	LR		99.2	97.4–100	S	
3	2018	52.2	21.9–76.0	R	Te, Tr, Co^f^	61.4	42.0–77.4	R		49.5	19.9–73.1	R	Te, Tr^f^
3	2021	21.5	−15.2–52.1	R	Te, Tr, Co^e^	89.6	79.4–96.6	R	Te, Tr^e^	85.2	66.5–95.1	R	
4	2022	NT				98.7	97.1–99.7	S		78.6	58.8–92.5	R	Te^e^
5	2018	59.2	38.2–76.4	R		99.6	99.2–99.8	S		67.8	41.9–86.9	R	
6	2022	NT				97.6	95.6–99.1	S		99.8	99.3–100	S	
7	2018	59.7	41.4–75.1	R		95.8	91.8–98.6	LR		90.9	84.3–96.0	R	
7	2022	NT				98.2	95.7–99.7	S		94.3	89.0–98.1	R	
8	2019	37.1	7.1–62.0	R		93.9	89.2–97.4	R		88.0	87.5–95.0	R	
9	2018	24.1	−1.3–46.4	R	Te, Tr^f^	90.7	84.0–95.8	R		92.0	84.6–97.3	R	
10	2018	82.2	71.4–90.7	R		100	-	S		82.8	72.7–90.9	R	
11	2018	31.0	−15.8–67.1	R		91.2	81.5–97.6	R		98.7	97.0–99.7	S	
12	2023	34.0	−1.8–63.1	R		99.9	99.9–100	S		97.6	94.8–99.4	S	
13	2018	79.1	56.7–94.1	R		98.8	97.3–99.8	S		96.7	93.9–99.0	S	
14	2023	NT				94	89.5–97.3	R	Te^e^	83.0	70.1–92.6	R	Te^e^
15	2019	26.5	−22.2–64.2	R		96.3	93.7–98.3	LR		81.3	68.4–91.3	R	
16	2018	52.7	24.5–75.1	R	Te, Tr^f^	99.4	98.7–99.8	S		92.6	83.7–98.3	R	
17	2018	87.2	82.5–91.2	R		96.6	93.6–98.7	LR		89.3	81.9–95.1	R	
18	2018	84.9	74.5–92.6	R		99.9	99.8–100	S		60.2	44.2–73.9	R	
19	2019	68.4	53.2–81	R		98.3	96.7–99.5	S		83.2	73.3–91.1	R	
20	2023	NT				84.7	70.5–94.7	R		88.5	81.6–93.9	R	
21	2018	26.8	−36.6–72.5	R	Te, Tr, Co^f^	98.7	97.0–99.7	S		99.5	98.9–99.9	S	
21	2022	NT				96.6	94.4–98.3	LR		95.2	92.1–97.7	LR	
22	2018	82.5	73.4–89.9	R		96.8	93.1–99.2	S		96.4	93.1–98.8	LR	
23	2022	NT				93.5	86.1–98.4	R	Te^e^	89.6	80.6–96.1	R	
24	2019	28.8	−16–63.9	R		96.6	91.5–99.5	S		85.7	71.3–95.6	R	

^a^*NT* Not tested

^b^*UI* Uncertainty interval

^c^*S* Susceptible, *R* Resistant, *LR* Low Resistant

^d^Te = *Teladorsagia circumcincta*, Tr = *Trichostrongylus* spp., Co = *Cooperia* spp

^e^ = Larval identification by PCR

^f^ = Larval identification by morphology

Only three farms were tested with moxidectin and all displayed resistance, with one classified as low resistant; FEC reductions ranged from 62.8 to 96.5%.

### Larval ID

Of the 77 FECRTs conducted, pre- and post-treatment larval identifications were performed for only 6 fenbendazole tests (5 farms), 3 levamisole and 3 ivermectin tests as shown in Table 2. In each case *Teladorsagia circumcincta* larvae were identified post-treatment. For all 6 fenbendazole tests, one levamisole and one ivermectin test *Trichostronglyus* spp. were additionally identified. *Cooperia* spp. were identified post-treatment for 3 fenbendazole tests (2 farms).

## Discussion

Few studies have attempted to quantify the extent of anthelmintic resistance in GIN on Irish sheep farms. The first comprehensive report in 2012 revealed widespread resistance to the benzimidazole and imidazothiazoles classes. However, resistance to ivermectin was not tested in vivo [4]. A study in 2013 targeted farms where the farmer suspected ivermectin resistance and found ivermectin-resistant *T. circumcincta* on 2 of the 4 farms tested. This species was subsequently demonstrated to be also resistant to fenbendazole and levamisole but not moxidectin [8]. Other reports from Ireland have relied on drench tests i.e. pooled faecal samples pre- and post-treatment [9]. However, while these tests can indicate treatment failure, they are not sufficient to declare resistance. Therefore, 77 FECRTs were conducted between 2018 and 2023 on 24 sheep farms to gather data on the current extent of anthelmintic resistance in GIN. Farms were included based on the willingness of the farmer to participate in the study and there was no prior knowledge on the resistance status of the farms, except those farms on which tests were repeated. However, farmers concerned about AR or who believed they had a resistance problem may have been more likely to self-select, which could have influenced the results.

Resistance to fenbendazole was found on all farms tested. Resistance to levamisole was found on 10 farms (42%) with a further 2 farms having conflicting results when the test was repeated in another year. Resistance to ivermectin was found on 17 farms (71%) with one additional farm showing susceptibility in an initial test but low resistance in a later year. This is the first report of the extent of ivermectin resistance on sheep farms in Ireland. Compared with previous data, albeit based on in vitro and pooled faecal samples rather than the gold-standard FECRT, it appears that resistance to ivermectin is increasing on Irish sheep farms. This may reflect increasing use of this class given previous reports to resistance to the benzimidazole and imidazothiazole classes. Moxidectin was tested on only 3 farms and resistance was found on all farms tested. These three farmers had requested that moxidectin be tested in addition to the other anthelmintics and may have suspected resistance to this drug.

One notable feature of this study is that of the 7 FECRTs repeated in another year, 3 gave conflicting results with susceptibility found in one test and low resistance in the other. The reason for this is unclear; however, the FECRT cannot reliably detect resistance when resistant phenotypes make up small proportion of the population (< 25%) (Martin et al., 1989). Indeed, it has previously been reported that one-third of FECRTs that reported susceptibility based on total egg count in fact contained resistant genera/species that could only be detected when treatment efficacy was calculated per genus/species (McKenna, 1997). Resistance is most difficult to diagnose when there is only a small reduction in efficacy because the resistant species is present at low frequency [11]. For 2 of the farms susceptibility was found in the initial test, with low resistance found subsequently. This could reflect the development of resistance in the interim or the importation of resistant worms. However, on the other farm low resistance was reported initially and susceptibility subsequently, which more likely reflects variations in the proportion of the resistant species in pre-treatment samples [13]. Therefore, a major limitation of this study is the low number of tests for which larval identification was performed. In order to calculate species-specific efficacy larval identification both pre- and post-treatment is required. In this study some tests had insufficient faecal material or larvae while in other cases the resources available to conduct the larval identification was limiting. Indeed, resistance overall is likely to be more widespread in sheep GIN than reported here due to the insensitivity of the FECRT. This underscores the importance of regular monitoring for treatment efficacy and/or resistance. It also demonstrates the necessity of combining the FECRT with identification of the GIN species present pre- and post-anthelmintic treatment [15]. This has traditionally been done by morphological identification of L3 to the genus level, an expensive and time-consuming process requiring significant expertise. However, the development of high-throughput molecular methods for species identification has made larval identification more accessible [1]. These methods have improved the ability of the FECRT to detect anthelmintic resistance and should be included in anthelmintic resistance testing protocols (Leathwick et al., 2025). Overall, anthelmintic resistance in GIN is widespread on sheep farms in Ireland and likely higher than reported here due to the low sensitivity of the FECRT. In the absence of a preventative biosecurity protocol AR can spread by animal movement; therefore, if purchasing sheep, it is important to reduce the risk of inadvertent introduction of resistant GIN**.** The use of the new actives, monepantel or derquantel, as a biosecurity treatment can help prevent the further spread of anthelmintic resistance. However, this must be combined with other sustainable parasite control principles such as avoidance of high-risk practices (e.g. dose and immediately moving to clean pasture), using an anthelmintic known to be highly effective on the farm and maintenance of an *in refugia* population of susceptible GIN.

## Data Availability

No datasets were generated or analysed during the current study.

## Abbreviations

AR: Anthelmintic resistance
GIN: Gastrointestinal nematode
FEC: Faecal egg count
FECR: Faecal egg count reduction
FECRT: Faecal egg count reduction test
WAAVP: World Association for the Advancement of Veterinary Parasitology
